# Patient-derived tissue cultures complement neurospheres for preclinical evaluation of AAV-mediated gene delivery in glioblastoma

**DOI:** 10.1007/s11060-026-05807-w

**Published:** 2026-09-22

**Authors:** Franziska Köhler, Katharina Hess, Cindy Koloske, Frank Gaunitz, Steffen K. Rosahl, Rüdiger Gerlach, Sonja Kallendrusch

**Affiliations:** 1 Institute of Anatomy, University of Leipzig, 04103 Leipzig, Germany; 2 Institute of Clinical Research and Systems Medicine, HMU Health and Medical University, 14467 Potsdam, Germany; 3 Department of Neurosurgery, University Hospital Leipzig, 04103 Leipzig, Germany; 4 Department of Neurosurgery, HELIOS Hospital Erfurt, 99089 Erfurt, Germany; 5 HMU Health and Medical University, 99084 Erfurt, Germany

**Keywords:** Glioblastoma, Tumor microenvironment, Gene therapy, Patient-derived models, Neurosphere cultures, Organotypic tissue slice cultures

## Abstract

Glioblastoma (GBM) is characterized by extensive intratumoral heterogeneity and a complex tumor microenvironment that complicate the preclinical evaluation of gene therapy vectors. We investigated how culture model, epidermal growth factor (EGF) supplementation, and adeno-associated virus (AAV) serotype influence vector-mediated gene delivery in patient-derived GBM models. Patient-derived neurospheres (PDNS) and patient-derived tissue slice cultures (PDTC) were transduced with AAV2 or AAV6 vectors encoding green fluorescent protein (GFP). Transduction was evaluated by live confocal imaging, quantitative PCR, flow cytometry, and immunofluorescence under EGF-containing and EGF-free culture conditions. In PDNS, AAV6 produced significantly greater GFP expression than AAV2 and demonstrated a dose-dependent increase in transduction at both 2 and 5 days after vector exposure. EGF supplementation altered transduction patterns and was associated with changes in PDNS growth and marker-defined stem-like cell populations. In contrast, PDTC preserved tissue architecture and stromal, vascular, and immune-associated compartments while revealing substantial interpatient variability in AAV-mediated transduction. AAV-mediated gene delivery in GBM is influenced by culture model, growth factor conditions, and capsid serotype. Patient-derived tissue slice cultures complement neurosphere models by preserving features of the native tumor microenvironment and may improve the preclinical evaluation of gene therapy vectors for GBM.

**Supplementary Information:**

The online version contains supplementary material available at https://doi.org/10.1007/s11060-026-05807-w.

## Introduction

Glioblastoma (GBM) remains the most aggressive primary malignant brain tumor in adults, characterized by diffuse infiltration, marked intratumoral heterogeneity, and almost universal recurrence despite multimodal therapy [1, 2]. A major obstacle to effective treatment is the coexistence of genetically and phenotypically distinct tumor cell populations embedded within a complex microenvironment that promotes therapeutic resistance and disease progression [3–5]. Although patient-derived in vitro models have improved the study of GBM biology, conventional cell culture systems only incompletely recapitulate the cellular diversity, extracellular matrix, and tissue architecture of native tumors. Consequently, there is growing interest in patient-derived neurospheres (PDNS) and organotypic patient-derived tissue slice cultures (PDTC) as complementary ex vivo models for translational research [6].

Adeno-associated virus (AAV) vectors are promising platforms for local gene delivery to GBM because they can support sustained transgene expression and can be administered directly to residual tumor tissue or the resection cavity [7]. However, successful translation of AAV-based strategies depends on accurate preclinical assessment of vector performance. AAV transduction is influenced by multiple biological factors, including capsid serotype, receptor availability, extracellular matrix composition, and the physiological state of the cell [8]. This is particularly relevant in GBM, where growth factor–driven stem-like programs and regional tumor heterogeneity may affect both vector entry and transduction efficiency. Among natural serotypes, AAV2 remains a reference vector for CNS gene delivery, whereas AAV6 has shown promising activity in glioma models [9]. Experimental evidence suggests that the epidermal growth factor receptor (EGFR) may contribute to AAV6 cellular entry, although its functional relevance in patient-derived models of GBM remains incompletely understood [10, 11]. This raises the possibility that vector internalization is linked to signaling pathways frequently dysregulated in GBM, making EGF supplementation a relevant experimental variable. Importantly, most comparisons of AAV capsids in glioma models so far have been performed in reductionist cell culture systems, where vector access, receptor availability, and cellular state may differ substantially from those in intact tumor tissue. Whether serotype-specific differences observed in PDNS cultures are maintained in microenvironment-preserving GBM tissue models has not been systematically investigated.

To evaluate these context effects under translationally relevant conditions, we compared AAV2 and AAV6 in two complementary patient-derived GBM models: PDNS and PDTC. PDNS provides a controlled system for assessing tumor cell-intrinsic permissiveness, whereas PDTC better preserve cytoarchitecture, the extracellular matrix, and non-neoplastic components of the GBM microenvironment [12]. We therefore hypothesized that AAV transduction would differ between PDNS and PDTC and that EGF supplementation would modify serotype-specific vector responsiveness, particularly in AAV6-treated cultures. Our aim was to determine how experimental model, growth factor conditions, and capsid serotype affect AAV transduction in patient-derived GBM and to evaluate the utility of these complementary ex vivo systems for preclinical assessment of gene therapy vectors.

**Fig. 1:**
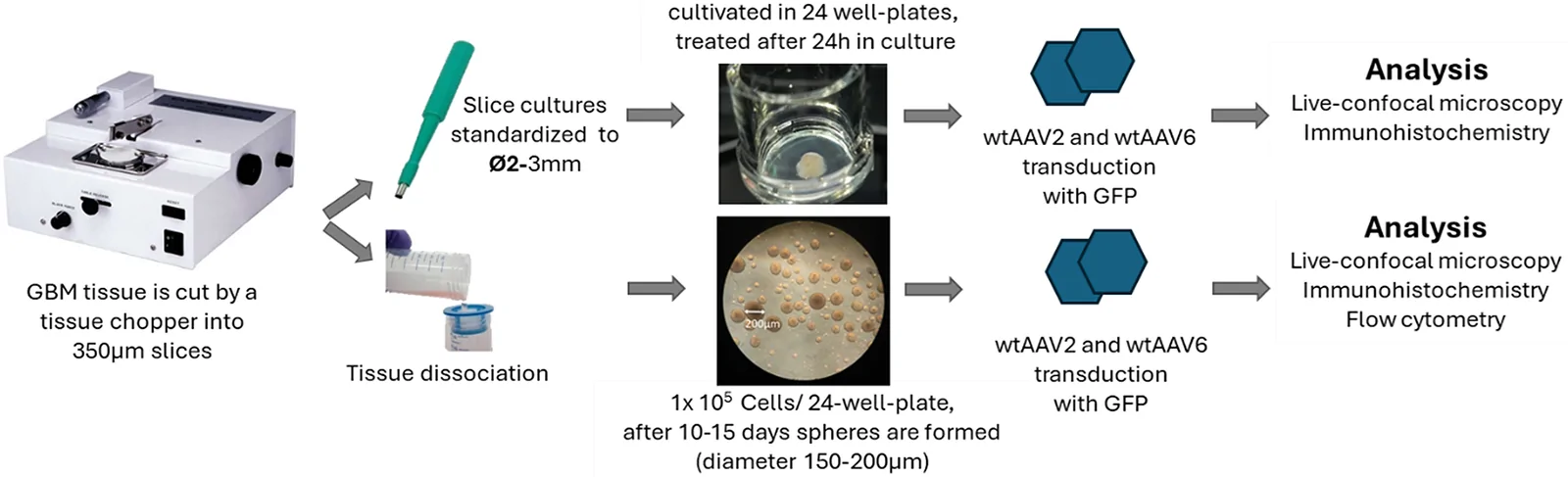
Experimental workflow for generating patient-derived GBM tissue slice cultures (PDTC) and neurosphere cultures (PDNS). Fresh tumor tissue was sectioned into 350 μm slices using a tissue chopper. Tissue punches (2–3 mm) were cultured on membrane inserts at an air–liquid interface to establish PDTCs. Remaining tissue was mechanically and enzymatically dissociated and cultured under serum-free conditions to generate PDNS. AAV vectors encoding GFP were added to the culture medium, and transduction was monitored by live confocal microscopy. At endpoint, cultures were analyzed by immunohistochemistry for GFP expression, stem cell markers, and microenvironmental cell populations to assess vector tropism and biological effects

## Methods

### Ethics statement

Tissue not required for routine neuropathological diagnosis was processed in accordance with the approval of the Ethics Committee of the Medical Faculty of Leipzig and Brandenburg. Written informed consent was obtained from all patients. The following procedure is also shown in Fig. 1. A total of 25 surgical tumor tissue samples were collected for this study. Eight samples were excluded because PDNS could not be successfully established. Four additional samples were excluded because the final histopathological diagnosis revealed a malignancy other than glioblastoma. The remaining 13 specimens were included in the study. According to the WHO 2021 classification, 12 tumors were IDH-wildtype glioblastomas, whereas one tumor was an IDH-mutated astrocytoma. Due to the limited availability of samples, the IDH-mutated astrocytoma was included only in the vector titration experiments and was not used for analyses characterizing glioblastoma biology. In two patients, non-neoplastic brain tissue (histologically confirmed) adjacent to the tumor became available to facilitate access to the tumor specimen. These tissues were established as PDTC and cultured and transduced with AAV vectors using the same protocol as for the corresponding tumor-derived slice cultures.

### Patient-derived tissue slice cultures

For the preparation of PDTC, fresh GBM tissue was cut into 350 μm slices using a tissue chopper (McIlwain TC 752; Campden Instruments, Lafayette, IL) and transferred to a Petri dish containing Minimum Essential Medium (MEM; Invitrogen). Using biopsy punches (Kai Europe, Solingen, Germany) with diameters of 2–3 mm, depending on the size of the original tissue sample, slices were transferred onto membrane inserts (Millipore Corporation, Billerica, MA) and cultured at an air–liquid interface in 24-well plates (TPP Techno Plastic Products AG, Trasadingen, Switzerland). Incubation was performed at 36 °C and 5% CO₂, and medium was changed every 48–72 h.

To ensure comparability with the PDNS model, several media established in the laboratory were compared with NeuroCult™-XF Proliferation Medium (Stemcell Technologies, Vancouver, Canada). A serum-containing medium consisting of MEM, 25% Hank’s Balanced Salt Solution with Ca^2+^ and Mg^2+^ (Gibco), 10% normal horse serum, 1% L-glutamine (200 mM stock solution, Braun; final concentration 2 mM), 1% glucose (45% stock solution, Braun; final concentration 0.45% [w/v]), and 1% penicillin/streptomycin (5000 U/mL penicillin stock, Sigma; final concentration 50 U/mL penicillin and 50 µg/mL streptomycin). In addition, a serum-free medium was evaluated consisting of MEM supplemented with 25% Hank’s Balanced Salt Solution (HBSS) containing Ca²⁺ and Mg²⁺ (Gibco), 10% glucose (5% stock solution, Braun; final concentration 0.5% [w/v]), 5% B-27™ supplement (Gibco), 1% L-glutamine (200 mM stock solution, Braun; final concentration 2 mM), 1% vitamin C (stock solution: 100 mg in 10 mL water; final concentration 0.1 mg/mL [100 µg/mL]), and 1% vitamin E (DL-α-tocopherol, Alfa Aesar; 1% stock solution; final concentration 0.01% [w/v]).and 1% penicillin/streptomycin (Sigma), as well as NeuroCult™-XF Proliferation Medium supplemented with epidermal growth factor (EGF; 20 ng/ml), fibroblast growth factor (FGF; 10 ng/ml), and heparin (2 ng/ml). In subsequent experiments, PDTCs were maintained exclusively in NeuroCult™ medium.

### Patient-derived neurosphere cultures

PDNS cultures were generated from tissue remnants remaining after PDTC preparation. Tissue was dissociated by incubation with Accutase at 37 °C for 30–60 min, followed by repeated trituration with a glass pipette. After dilution with MEM, filtration through a 40 μm mesh filter, centrifugation, removal of Accutase-containing medium, and resuspension in NeuroCult™ medium, a single-cell suspension was obtained. Cells were stained with trypan blue and counted using a Neubauer counting chamber. Subsequently, 100,000 cells in 500 µl medium were seeded per well of a 24-well plate and incubated at 36 °C and 5% CO₂. Medium was changed every 48–72 h. After 10–15 days, PDNS reached a diameter of 100–150 μm and were used for experiments.

For passaging of PDNC, the PDNC containing medium was pooled into a 50 ml Falcon tube. Wells were rinsed again with medium or, if necessary, incubated for a few minutes with 200 µl Accutase at 37 °C to detach adherent PDNS from the plate surface. Dissociation, resuspension in culture medium, cell counting, and reseeding into new 24-well plates were performed analogously to the initial preparation.

### AAV transduction

The AAV vectors used for transduction contained GFP in the transgene cassette. Successful transduction was directly detectable as green fluorescence by confocal microscopy of viable tissue. Self-complementary vector constructs were used, in which the vector genome folds back on itself and hybridizes intramolecularly, resulting in double-stranded DNA and enabling early transgene expression approximately 4 h after gene transfer. AAV vectors were obtained from the laboratory of Hildegard Büning and from signaGen Laboratories (cat. No. AAV2 and AAV6) [13].

AAV6 uses EGFR as a co-receptor for transduction. Because cultivation in EGF-containing medium may alter EGFR density, selected experimental conditions of PDTC were maintained in NeuroCult™ medium without EGF [11]. Since successful growth of PDNS cannot be ensured in EGF-free medium, PDNS were initially cultured in EGF-containing medium as described above. Once PDNS reached the desired size, they were transferred to EGF-free medium for 3 days. Vector incubation was subsequently performed in EGF-free medium. For this purpose, the desired number of PDNS was transferred under a stereomicroscope from culture plates into wells of a 96-well plate. The corresponding vectors were then added to the medium of selected spheroids and tumor slice cultures. Following vector application, no further medium changes were performed to avoid altering vector concentration. On day 3, 50 µl of vector-free medium was added to compensate for evaporation. At defined time points, live imaging was performed using confocal microscopy. Slice cultures derived from primary patient material were treated with vectors after 24 h in culture and incubated for an additional 5 days.

### Live imaging and quantification

Live imaging was performed using a confocal laser scanning microscope (FV1000, Olympus). Successful AAV-mediated gene transfer was assessed by GFP fluorescence. On the final day of incubation, propidium iodide (1 µg/mL) was added to visualize non-viable cells. For each PDNS, three optical sections were acquired: one through the center of the spheroid and two additional sections located 20 μm above and below the center. For PDTC, one image was acquired at the tissue periphery adjacent to the air–liquid interface and one in the central region of the slice. To determine the dose–response relationship, AAV6 vectors were titrated in PDNS. Three spheroids (100–150 μm diameter) were transferred to individual wells of a 96-well plate and exposed to 10⁶, 10⁷, 10⁸, or 10⁹ vector particles. Live imaging was performed on days 2 and 5 after vector application using the imaging protocol described above. To ensure comparability of fluorescence intensity in the GFP channel, a constant exposure time was maintained for all images. GFP signals were detectable in slice cultures by day 2 post-transduction. Quantitative fluorescence analysis was performed using ImageJ [14]. For each optical section, the cross-sectional area of the spheroid was manually outlined, the fluorescence intensity within this region of interest was measured, and the value was normalized to the respective area. This yielded the mean green fluorescence per spheroid cross-sectional area. A dose of 4 × 10^7 particles per well was applied to slices with a diameter of 3 mm. This resulted in a particle-to-cell ratio approximately comparable to that used for PDNS cultures transduced with 10^7 particles. In addition, this represents a realistic tissue concentration that may be achievable in a clinical setting.

### Quantitative PCR

To complement fluorescence-based assessment of AAV-mediated gene delivery, cell-associated vector genome abundance was quantified by qPCR in PDNS as previously described [15]. After 5 days of vector exposure, PDNS were harvested, washed, and trypsinized to minimize detection of vector particles remaining in the culture medium or associated with the spheroid surface. Total DNA was extracted using the DNeasy Blood & Tissue Kit (QIAGEN, Hilden, Germany). Vector genomes were quantified using GFP-specific primers and normalized to the human single-copy reference gene PLAT. The following primers were used: GFP forward, 5′-GCT ACC CCG ACC ACA TGA AG-3′; GFP reverse, 5′-GCT CAT GCC GAG AGT GAT CC-3′; PLAT forward, 5′-ACC TAG ACT GGA TTC GTG-3′; and PLAT reverse, 5′-AGA GGC TAG TGT GCA T-3′. Vector genome abundance was calculated by normalization of GFP to PLAT and expressed as vector genome copies per cell.

### Flow cytometry

To assess colocalization of selected stem cell markers with GFP-positive cells, AAV-treated PDNS were processed for flow cytometric analysis. Cells were stained for CD133, SOX2, and Nestin, and native GFP fluorescence was detected. Nine spheroids per condition (each 100–150 μm diameter) were incubated with the respective vectors for 5 days. Spheroids were then dissociated using Accutase and washed several times with FACS buffer consisting of PBS, 2% fetal calf serum (Gibco), and 2 mmol EDTA. Non-viable cells were labeled using a fixable viability dye (LIVE/DEAD™ Cell Stain Kit, Thermo Fisher Scientific, Cat. No. L34957). To prevent nonspecific antibody binding to Fc receptor–expressing cells, samples were treated with Fc block (eBioscience, 14-9161-73). Cells were then incubated with anti-CD133-PE/Cy7 (Biolegend, 372809) and fixed with Cytofix (BD Biosciences, 554723). For intracellular and nuclear antigens, cells were permeabilized with Perm/Wash buffer and incubated with anti-Nestin-PE (BioLegend, B269407) and anti-SOX2-Pacific Blue (BioLegend, 656111). Samples were analyzed on an LSR II flow cytometer. FACSDiVa 6.1.3 software was used for acquisition and initial analysis, and FlowJo v10.2 for documentation and analysis.

### Histology

For histological processing, samples were fixed in 4% paraformaldehyde. PDTCs were transferred into embedding cassettes and dehydrated, followed by paraffin embedding. Due to their small size, PDNS were first embedded in an agar block. For this purpose, a 1% agarose gel was prepared and kept warm (36 °C) on a heating plate. Under a stereomicroscope, fixed spheroids were transferred from 96-well plates into cryo-embedding molds, excess medium was removed, and the molds were filled with agarose. After gel solidification, blocks were processed in the same manner as PDTCs. Tissue sections were cut at a thickness of 7 μm. For immunofluorescence staining, samples were incubated overnight with primary antibodies (*stem cell marker*: SOX2, Neuromics, GT15098 (1:200); Nestin, Merck, ABD69 (1:1000); OLIG2, Epitomics, AC-0106RUO (1:200); CD44, Cell Signaling Technologies, 156-3C11 (1:400); Survivin, Cell Signaling Technologies, 2808 (1:500) *astrocyte marker*: GFAP, DAKO, Z0334 (1:500); *AAV transduction*: GFP, Synaptic Systems, 132005 (1:400); *apoptosis and proliferation*: Cleaved PARP1, abcam, ab32064 (1:100), Ki67, DCS, KI681C01 (1:200)). After washing, samples were incubated with appropriate fluorophore-conjugated secondary antibodies. Finally, nuclei were stained with DAPI, and slides were mounted using Fluorescence Mounting Medium (Agilent Dako, S302380).

### Statistical analysis

Histological quantification was performed using a previously established semi-automated image analysis pipeline [16]. For each section, images were acquired from three representative regions. Autofluorescence correction, thresholding, and binary conversion were performed in ImageJ, followed by pixel-based colocalization analysis with DAPI to calculate fractions of proliferative and apoptotic cells as well as stem cell marker and GFP fractions. Given the high biological heterogeneity and limited sample size (*n* = 3–8 tissue specimens), non-parametric statistical tests were employed for all comparisons. The Kruskal–Wallis test was used to assess differences across multiple independent groups, followed by Dunn’s post hoc test for pairwise comparisons. In this study, n represents one tissue specimen, consisting of three individual PDNS or three tumor slice cultures derived from that specimen. Exact p-values are reported where possible, and a p-value ≤ 0.05 was considered statistically significant. All analyses were performed using GraphPad Prism 10.0.

## Results

### Culture conditions preserving tissue architecture

**Fig. 2:**
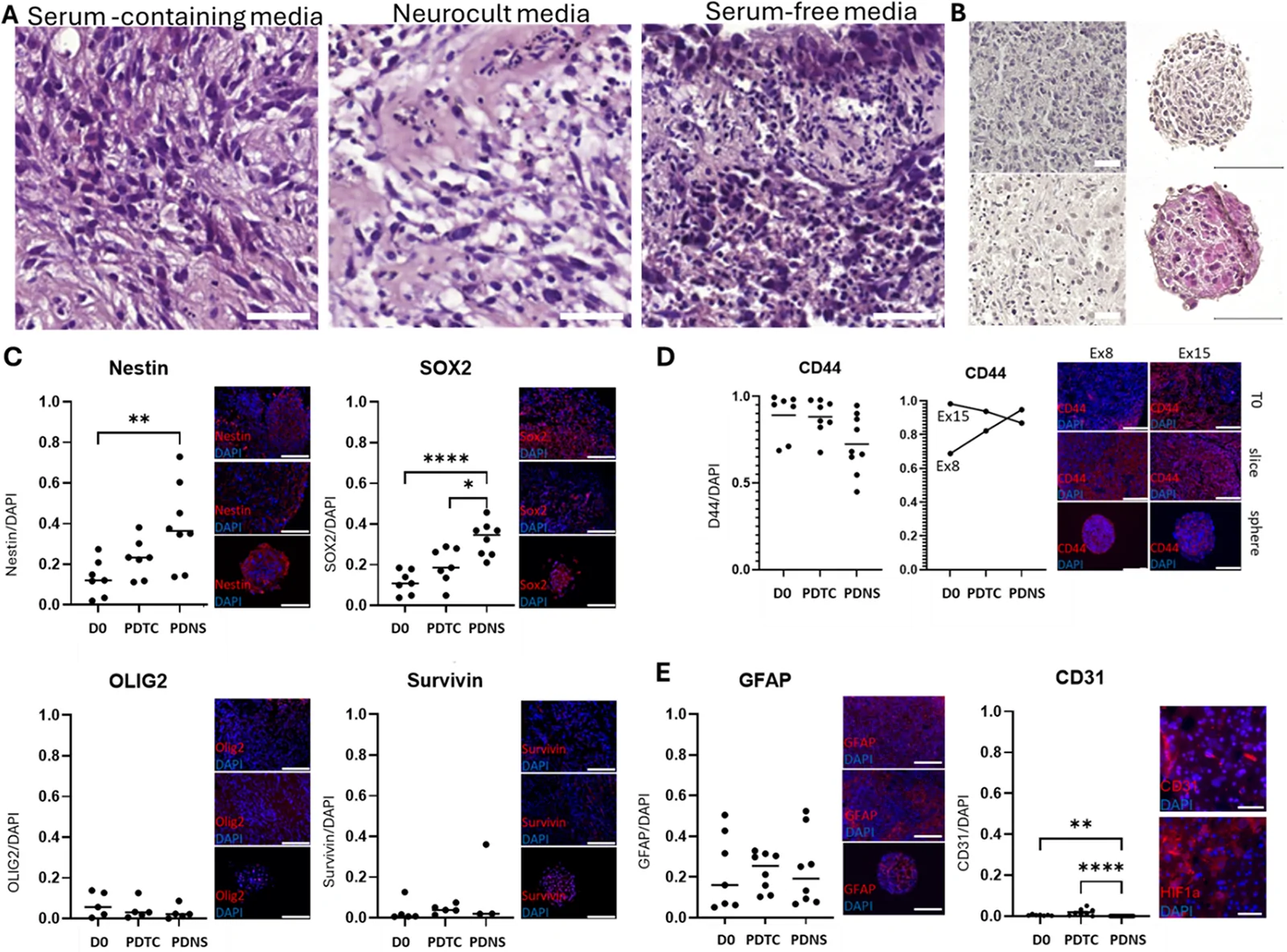
Histological characterization of PDTC and PDNS (**A**) Representative hematoxylin and eosin (H&E) staining of GBM PDTCs cultured under different media conditions to evaluate preservation of tissue morphology and architecture (scale bar = 50 μm). Organotypic hippocampal slice culture medium and serum-free NeuroCult medium maintained tissue integrity most effectively, whereas other serum-free media resulted in progressive loss of structural organization. (**B**) Representative images illustrating morphological differences between PDTCs and matched PDNS generated from two independent patient specimens (upper and lower rows, respectively, Neurocult media). PDTCs preserved native tissue architecture and cellular heterogeneity, whereas PDNS formed compact multicellular spheroids with reduced structural complexity (scale bar = 100 μm). (**C**) Quantification of stem cell markers in native tumor tissue, PDTCs, and PDNS (*n* = 4 patients, Neurocult media). Expression of stem cell-associated markers (Survivin, Olig2, SOX2, Nestin, and CD44) was compared between native tissue, PDTCs, and PDNS. (**D**) CD44 expression differed significantly between native tissue and PDNS, although substantial inter-patient heterogeneity was observed. (**E**) GFAP expression was preserved across culture conditions, whereas CD31-positive endothelial cells were largely absent in PDNS. Representative immunohistochemical staining for HIF1α is shown in PDTC. Statistical analysis was performed using the Kruskal–Wallis test followed by Dunn’s multiple-comparison test (*p < 0.05, **p < 0.01, ****p < 0.0001)

Based on previous reports demonstrating impaired preservation of stem-like tumor cell populations under serum-containing conditions, we evaluated serum-free alternatives for GBM tissue slice cultures. We therefore evaluated serum-free alternatives, including a serum-free variant of the established medium and NeuroCult™ medium, which is also used for neurosphere cultures. After 7 days in culture, all conditions showed reduced proliferation and increased apoptosis relative to native tissue, but only the serum-containing medium induced significant changes (suppl. Figure 1). Blinded pathological assessment demonstrated that NeuroCult™ medium preserved tissue architecture and histopathological characteristics more effectively than the alternative serum-free formulation (Fig. 2A). Clear culture-associated adaptations were visible in direct comparison with native tissue.

Despite culture-associated changes, characteristic morphological features of the original tumors remained recognizable in both PDTC and PDNS. As shown in Fig. 2B, fibrillary tumor architecture was preserved across both culture systems. Similarly, gemistocytic features, including enlarged cell bodies, abundant cytoplasm, and eccentrically positioned nuclei, were retained in both models. These findings indicate that key patient-specific histomorphological characteristics can be maintained under both culture conditions. Based on the superior preservation of tissue architecture and tumor morphology, NeuroCult™ medium was selected for all subsequent slice-culture experiments.

### Cellular composition

Expression profiles of key cellular and stem cell-associated markers were compared between PDTC, matched PDNS, and the corresponding native tumor tissue. Markers reflecting stromal, vascular, and immune components revealed pronounced differences between the two culture systems. CD68-positive cells were rarely detectable in PDNS and lacked organotypic distribution, whereas CD31 was completely absent. Hypoxia-inducible factor 1α (HIF1α) was only weakly expressed in PDNS and was restricted to spheroids larger than 200 μm in diameter. In contrast, PDTC retained expression of all investigated markers, including immune, vascular, and hypoxia-related markers, in an organotypic pattern closely resembling the corresponding native patient-derived tumor tissue (Fig. 2C). Expression of OLIG2, survivin, and GFAP was preserved in both PDTC and PDNS and did not differ from the corresponding uncultured, native patient tissue. In contrast, the stemness-associated markers SOX2 and Nestin were increased in spheroid cultures (mean SOX2 positive cell fraction: CTR 0.114 vs. PDNS 0.328 *p* ≤ 0.0004 and mean Nestin positive cell fraction: CTR 0.132 vs. PDNS 0.393 *p* ≤ 0.0045, respectively) whereas CD44 expression was reduced compared with PDTCs and native tissue (mean CD44 positive cell fraction: CTR 0.892 vs. PDNS 0.725 *p* ≤ 0.0125). However, CD44 was also examined by pathological scoring, and high individual adaptations were observable (Fig. 2C and suppl. Figure 2). Further, two patient specimens were studied to investigate the influence of serum during transportation of the tissue, but no alterations of the stem cell population could be observed in PDTC when cultivated in NeuroCult media, consequently (data not shown).

### AAV transduction

**Fig. 3:**
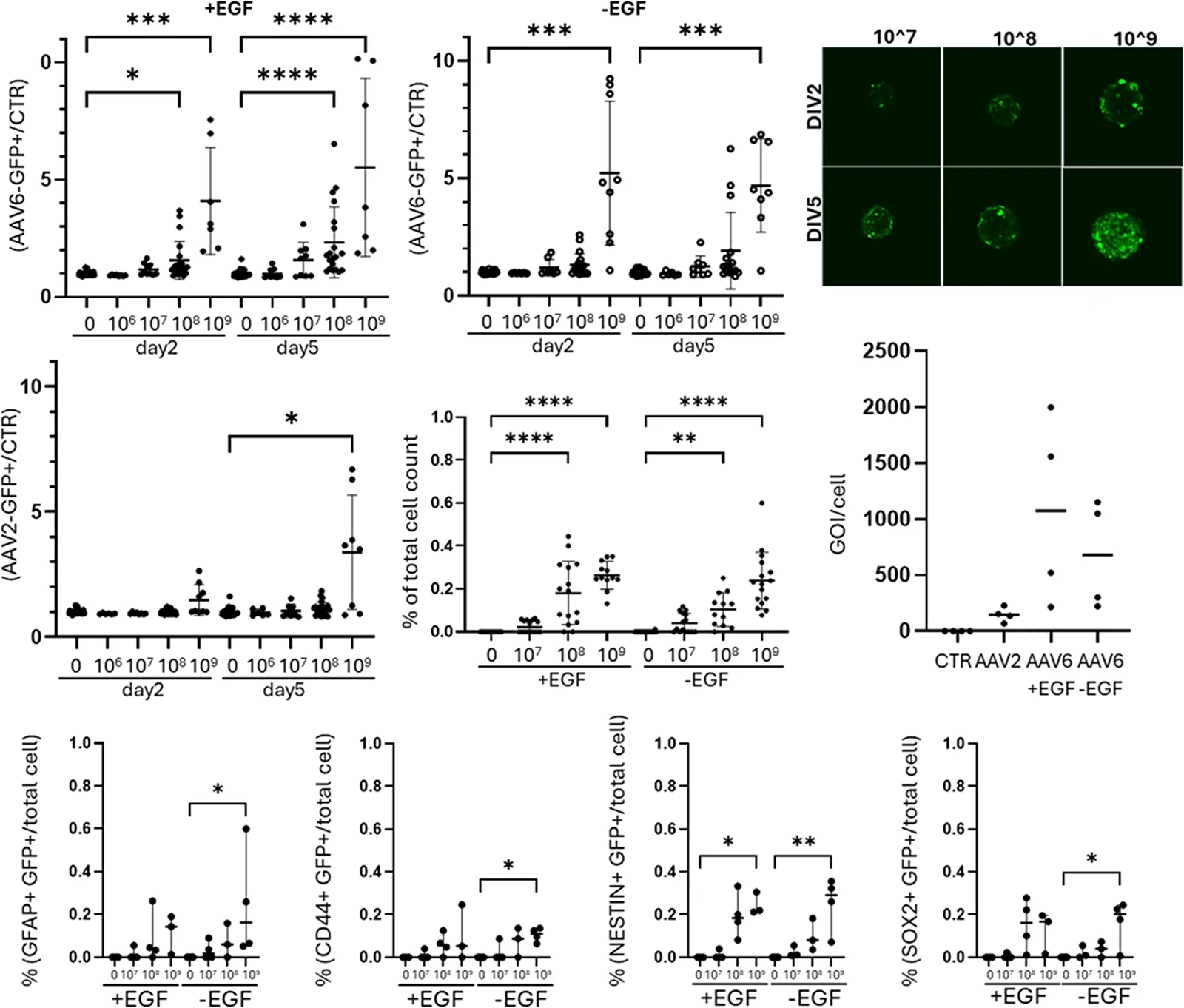
AAV2 and AAV6 transduction of patient-derived neurosphere cultures (PDNS) under EGF-supplemented and EGF-deprived conditions. (**A**) Dose-response analysis of AAV-mediated transduction in PDNS. Spheroids derived from at least three independent GBM specimens were transduced with AAV2 or AAV6 vectors at doses ranging from 10^6^ to 10^9^ vector particles per well. GFP expression was monitored by live confocal microscopy on days 2 and 5 post-transduction. Quantification of GFP fluorescence demonstrated a dose-dependent increase in transduction efficiency for both serotypes. For AAV6, significant increases in GFP signal were observed at 10^8^ particles under EGF-supplemented conditions, whereas AAV2 reached significance only at 10^9^ particles on day 5. Representative confocal images of AAV6-transduced spheroids cultured in the presence of EGF are shown. To validate live-imaging results and determine cellular tropism, spheroids were fixed on day 5, embedded, and analyzed by immunofluorescence staining for GFP. (**B**) PLAT-normalized AAV vector genome abundance in PDNS 5 days after treatment with 10⁸ vector particles is shown (*n* = 4). (**C**) Cell type–specific analysis of AAV transduction in PDNS. Co-immunofluorescence staining for GFP and the lineage-associated markers GFAP, CD44, Nestin, and SOX2 was performed to identify transduced cellular populations. In EGF-deprived media, increased proportions of GFP-positive cells were observed across multiple marker-defined populations. In contrast, under EGF-supplemented conditions, the strongest enrichment of GFP expression was detected within the Nestin-positive population. Statistical analysis was performed using the Kruskal–Wallis test followed by Dunn’s multiple-comparison test (*p < 0.05, **p < 0.01, ***p < 0.001,****p < 0.0001)

A dose-dependent increase in AAV-mediated GFP expression was observed in PDNS, with differences between serotypes and culture conditions (Fig. 3). AAV2 produced a modest increase in GFP fluorescence, reaching significance only after 5 days at the highest vector dose of 10⁹ particles (MFI 3.380, *p* ≤ 0.0211). In contrast, AAV6 induced significantly increased GFP fluorescence already at day 2 at both 10⁸ and 10⁹ particles (MFI 1.562, *p* ≤ 0.0334 and MFI 4.091, *p* ≤ 0.0005, respectively). This effect increased further by day 5 (10⁸ particles: MFI 2.328, *p* ≤ 0.0001; 10⁹ particles: MFI 5.521, *p* ≤ 0.0001). In the absence of EGF, AAV6-mediated GFP expression showed less temporal variation, with significant GFP expression detected at both day 2 and day 5 following vector application (MFI 5.217 and 4.685, respectively; both *p* ≤ 0.0002).

To complement the fluorescence-based assessment of AAV-mediated gene delivery, intracellular vector genome abundance was quantified by qPCR after 5 days of treatment with 10⁸ vector particles (*n* = 4). GFP vector genome copy numbers were normalized to the human single-copy reference gene PLAT to account for differences in cellular DNA content between samples. PLAT-normalized vector genome abundance was negligible in untreated controls (0.10 ± 0.10) and increased to 146.7 ± 65.4 following AAV2 treatment. Substantially higher vector genome abundance was detected following AAV6 treatment, with 1,074.0 ± 843.9 in the presence of EGF and 680.8 ± 486.9 in the absence of EGF (PLAT-normalized mean ± SD; *n* = 4). Thus, molecular quantification supported the fluorescence-based findings, demonstrating substantially greater vector genome abundance following AAV6 compared with AAV2 treatment. A higher mean vector genome abundance was also observed for AAV6 under EGF-containing compared with EGF-free conditions, although considerable inter-sample variability was present.

An additional independent image-based readout, the GFP-positive area was quantified relative to the total cellular area after 5 days. No GFP signal was detected in untreated controls. Consistent with the MFI analysis, AAV6-mediated GFP expression was greater under EGF-containing conditions than under EGF-free conditions. At 10⁸ particles, the GFP-positive area fraction was 0.180 with EGF (*p* ≤ 0.0001) compared with 0.104 without EGF (*p* ≤ 0.0012); at 10⁹ particles, the corresponding values were 0.264 (*p* ≤ 0.0001) and 0.238 (*p* ≤ 0.0001), respectively. These findings supported the dose-dependent and EGF-associated differences identified by fluorescence-intensity analysis. In two PDNS experiments, GFP expression was followed for up to 14 days after vector exposure (suppl. Figure 3). In the absence of EGF, AAV6-mediated GFP expression remained comparatively stable throughout the observation period, with values ranging from 0.059 to 0.080 between days 2 and 14. In EGF-containing medium, AAV6 initially produced higher GFP expression but showed a progressive decline from 0.171 at day 2 to 0.029 at day 14. AAV2 reached a similar level of GFP expression but remained stable only until day 7 (data not shown). Quantitative analysis of propidium iodide staining in two experiments did not reveal significant differences in the proportion of non-viable cells between the investigated culture conditions (suppl. Figure 3). Analysis of marker-defined cellular populations further demonstrated an influence of EGF conditions on the distribution of AAV6-associated GFP expression. Following treatment with 10⁹ AAV6 particles in the absence of EGF, significant GFP-positive fractions were detected among GFAP-positive (0.322, *p* ≤ 0.0189), CD44-positive (0.212, *p* ≤ 0.0248), Nestin-positive (0.269, *p* ≤ 0.0092), and SOX2-positive cells (0.291, *p* ≤ 0.0288). In the presence of EGF, only the Nestin-positive population showed a significant GFP-positive fraction at this vector dose (0.259, *p* ≤ 0.0395). To further explore whether GFP expression was associated with specific cellular phenotypes rather than solely reflecting their relative abundance, flow-cytometric data from two independent experiments were analyzed descriptively (suppl. Figure 3). In the pooled dataset, GFP positivity was most pronounced among SOX2-positive cells (40.8% vs. 7.2% in SOX2-negative cells) and CD133/Nestin/SOX2 triple-positive cells (41.0% vs. 7.5% in non-triple-positive cells). GFP positivity was also higher among Nestin-positive than Nestin-negative cells (21.7% vs. 1.2%) and among CD133-positive than CD133-negative cells (22.0% vs. 7.0%). Because only two independent experiments were available, due to the low cellular viability in the third experiment, these findings were considered exploratory and no inferential statistical analysis was performed.

Together, the immunohistological and flow-cytometric findings indicate an association between cellular phenotype and AAV6-mediated GFP expression, but do not establish preferential vector uptake by a specific GBM cell state. Spatially, GFP-positive cells were predominantly located in peripheral regions of the spheroids, although individual GFP-positive cells were also detected in central regions.

### Patient-derived tissue culture

**Fig. 4:**
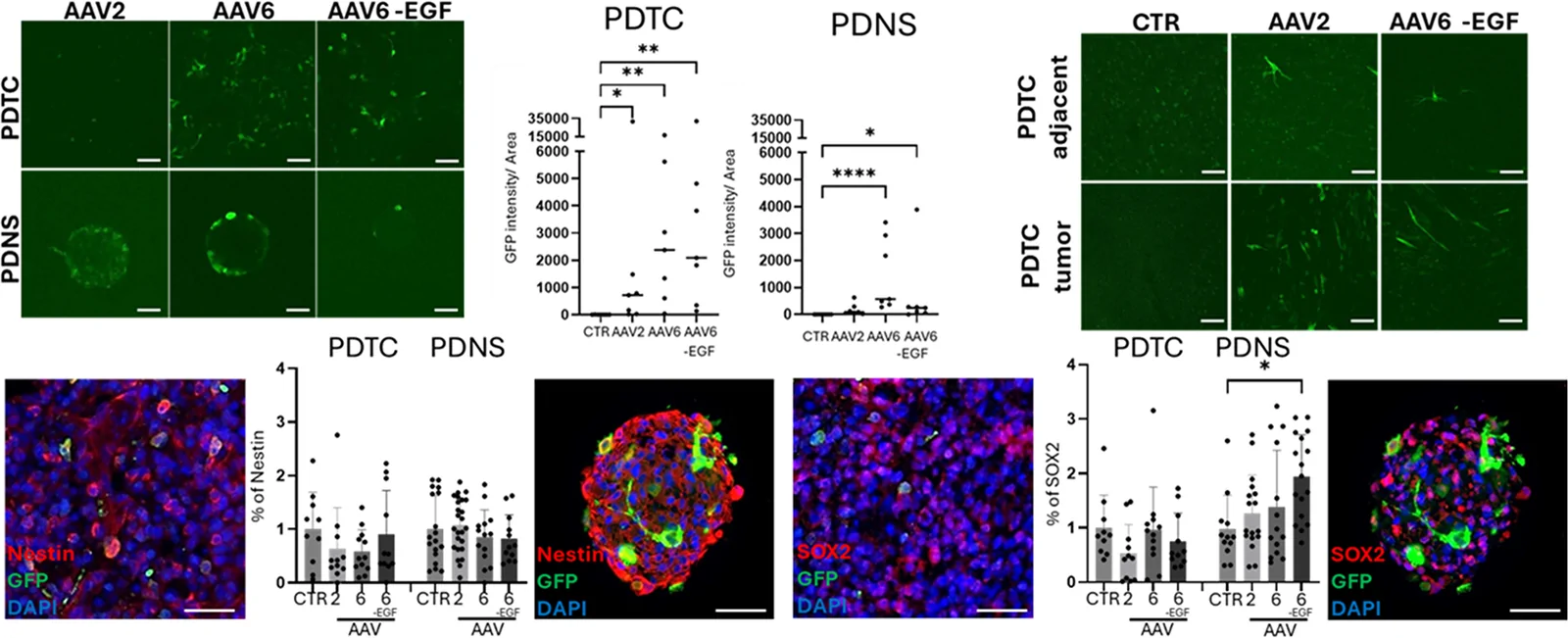
Comparison of AAV-mediated transduction and stem cell marker expression in patient-derived tissue cultures (PDTCs) and neurosphere cultures (PDNS). (**A**) Quantitative comparison of AAV transduction in matched PDTC and PDNS generated from seven independent GBM specimens (shown is the median). GFP fluorescence intensity was used as a readout of AAV-mediated gene delivery following treatment with AAV2 or AAV6 under EGF-supplemented and EGF-deprived conditions. PDTCs exhibited greater inter-patient variability than PDNS across all experimental conditions. In PDTCs, both AAV2 and AAV6 significantly increased GFP expression compared with control cultures. In contrast, only AAV6 induced a significant increase in GFP fluorescence in PDNS, whereas AAV2 did not differ significantly from control conditions. EGF supplementation had minimal effects on transduction efficiency in PDTCs, while omission of EGF reduced GFP expression in PDNS, particularly following AAV6 transduction. (**B**) GFP expression could also be observed in PDTC of non-malignant origin in both serotypes investigated in two independent tissue specimen. (**C**) Expression of stem cell-associated markers following AAV transduction in PDTCs and PDNS. Nestin expression remained stable across culture systems and treatment conditions. In contrast, SOX2 expression exhibited culture- and treatment-dependent variability. A significant increase in SOX2 expression was observed in PDNS transduced with AAV6 under EGF-deprived conditions compared with untreated controls, whereas no significant alterations were detected in PDTCs. These findings indicate that SOX2 expression varied with culture and treatment conditions in PDNS, whereas no significant changes were detected in PDTCs. Statistical analyses were performed using the Kruskal–Wallis test followed by Dunn’s multiple-comparison test (*p < 0.05, **p < 0.01, ****p < 0.0001). Individual data points represent independent patient specimens

AAV-mediated GFP expression in PDTCs varied substantially in magnitude and spatial distribution between patient specimens. Because tissue availability was limited, vector titration could not be performed in PDTCs, and the number of vector particles applied was estimated based on the cellular content of the tissue. Direct comparison of GFP fluorescence between PDTCs and matched PDNS should nevertheless be interpreted cautiously because the two models differ in cellular composition, tissue architecture, and vector exposure, like tissue penetration. While PDNS are in direct contact with the media, PDTC must actively take the media up through a membrane. Marked differences in fluorescence intensity were observed both within individual patient samples and between different patients (Fig. 4). In some experiments, no successful transduction could be detected under any condition. Transduction in PDTC was most intense after 5 days and was therefore used for comparison (data not shown). Direct comparison of fluorescence intensity showed high transduction in PDTC (AAV2, 4989 MFI, *p* ≤ 0.0367; AAV6, 4232 MFI, *p* ≤ 0.0015; AAV6 without EGF, 6448 MFI, *p* ≤ 0.0010) relative to PDNS (AAV2, 180 MFI, *p* ≤ 0.0563; AAV6, 1460 MFI, *p* ≤ 0.0001; AAV6 without EGF, 695 MFI, *p* ≤ 0.0321) however, inter-patient variability was also greater in PDTC. Because transduction was successful in PDTC, two adjacent non-tumor tissues were also cultured and treated. Both tissues showed transduction of single cells, although these cells were not further characterized (Fig. 4B).

Finally, AAV serotype and EGF appeared to influence stem cell-marker expression in a culture-dependent manner. While Nestin expression remained stable in both PDTC and PDNS, SOX2 expression showed non-significant alterations in PDTC (mean SOX2 expression normalized to control AAV2 0.531). In spheroid cultures, SOX2 expression was non-significantly increased after AAV2 (mean SOX2 expression normalized to control 1.270) and AAV6 (mean SOX2 expression normalized to control 1.387) treatment and significantly increased after AAV6 treatment without EGF supplementation (mean SOX2 expression normalized to control 1.936, *p* ≤ 0.0196). CD44 did not show significant changes after serotype supplementation in either culture system, although this was only evident when spheroids without passaging were considered (suppl. Figure 4).

## Discussion

This study demonstrates that the experimental model substantially influences the evaluation of AAV-mediated gene delivery in GBM. By directly comparing PDNS with PDTC, we found that vector-associated GFP expression, spatial distribution, and cell-state-associated responses differed depending on culture method, growth factor conditions, and capsid serotype. These findings underscore that simplified in vitro systems do not fully predict vector performance in patient-derived tissue and highlight the importance of selecting biologically relevant model systems for preclinical evaluation of gene therapy strategies [6, 17, 18]. A major strength of PDTCs was their ability to preserve key features of the native GBM microenvironment. Compared with serum-containing conditions, serum-free culture media better maintained tissue architecture and marker expression, supporting previous reports that defined culture conditions minimize culture-induced alterations while preserving clinically relevant tumor characteristics [3, 19]. Although long-term preservation of tissue viability and function remains to be established, these findings support the use of serum-free organotypic cultures for short-term translational studies.

PDTC retained immune [20], vascular, and hypoxia-associated markers, whereas these compartments were largely absent or functionally diminished in PDNS. These observations are consistent with the well-documented loss of tumor microenvironment compartments in spheroid cultures [17]. Our findings therefore suggest that PDNS and PDTCs may serve distinct purpose during preclinical AAV evaluation. PDNS provide an intermediate patient-derived model suited to confirming vector performance and investigating tumor-cell-state-dependent effects following initial screening in established cell lines. Selected candidates could subsequently be evaluated in PDTCs from multiple patients, allowing assessment of vector performance in the context of preserved tissue architecture, cellular heterogeneity, and non-neoplastic components of the tumor microenvironment. PDTCs may therefore be particularly informative for evaluating intra-tumoral distribution, patient-to-patient variability, and GFP expression in non-tumor cell populations. Importantly, our data do not demonstrate that both models are required for preclinical AAV development or that either model predicts in vivo performance. Rather, they indicate that model selection influences the apparent performance and biological interpretation of AAV-mediated gene delivery and should therefore be guided by the specific experimental question.

Consistent with this structural divergence, stemness-associated markers exhibited model-dependent regulation. SOX2 and Nestin were enriched in PDNS relative to PDTC and native tissue, reflecting the well-described selection pressure for stem-like tumor cell populations under sphere-forming conditions [21, 22]. In contrast, CD44 expression was reduced in PDNS, consistent with a relative depletion of mesenchymal-like states in simplified culture systems [23, 24]. These findings reinforce the concept that GBM stem-like states are not fixed entities but dynamically regulated transcriptional programs shaped by extracellular cues, including growth factor signaling, oxygen availability, and matrix interactions. Therefore, the differences observed between PDNS and PDTCs may also be adaptations introduced during culture. Establishment and expansion of PDNS impose selective conditions that may enrich particular tumor-cell states and reduce representation of the cellular heterogeneity present in the original tumor. In contrast, PDTCs require substantially less cellular selection and initially preserve the spatial organization and cellular composition of the resected tissue, although ex vivo culture may itself induce time-dependent adaptations. Consequently, model-dependent differences in AAV-mediated gene delivery cannot necessarily be attributed to intrinsic biological properties of the originating tumors alone.

Within PDNS, AAV-mediated GFP expression increased with vector dose and differed between serotypes. AAV6 produced greater GFP expression than AAV2, consistent vector genome quantification and with previous studies demonstrating enhanced AAV6 activity in glioma models [9, 11, 25]. EGF supplementation further modified vector-associated GFP expression, indicating that growth factor conditions influence vector responsiveness. Although EGFR has been proposed to facilitate AAV6 cellular entry, the present study was not designed to determine the underlying mechanism. EGF supplementation may alter multiple biological properties relevant to vector performance, including proliferation, differentiation state, receptor expression, and intracellular trafficking [11, 26]. Notably, the influence of EGF supplementation differed substantially between the two patient-derived models. The pronounced EGF-dependent differences observed in PDNS were considerably attenuated in PDTCs, suggesting that the effect of growth factor supplementation is strongly dependent on culture context. PDNS consist of tumor cells selected and maintained under sphere-forming conditions and may therefore undergo more pronounced shifts in proliferation, differentiation state, or cellular composition in response to EGF withdrawal. In PDTCs, tumor cells remain embedded within their extracellular matrix and surrounded by multiple tumor-associated cell populations that may provide additional paracrine and matrix-derived signals, thereby reducing dependence on exogenous EGF. Thus, the strong EGF effect observed in PDNS should not necessarily be interpreted as a general property of patient-derived GBM tissue but may partly reflect adaptation to neurosphere culture conditions. Whether differences in EGFR abundance or signaling contribute to these observations remains to be determined.

Exploratory flow-cytometric analysis further suggested that AAV6-mediated GFP expression differed across marker-defined cellular states, with higher GFP positivity observed among Nestin-, CD133-, and particularly SOX2-positive populations. Because only two independent experiments were available, these findings remain descriptive and do not establish preferential vector uptake or a specific mechanism of cell-state-dependent AAV responsiveness. As differences in GFP positivity within marker-defined populations cannot be separated from changes in the abundance or composition of those populations. The observed associations may therefore reflect differences in AAV-mediated gene delivery, cell-state composition, cell survival, or a combination of these factors.

Accordingly, the present data support an association between marker-defined cellular states and AAV6-mediated GFP expression but do not establish preferential vector uptake, cellular tropism, or a specific molecular determinant of AAV6 susceptibility. Differences in vector entry, intracellular processing, cell survival, promoter activity, and population composition may all contribute to these observations [27–33].

Spatial analysis revealed a predominantly peripheral distribution of GFP-positive cells in PDNS spheroids, with occasional penetration into central regions. This pattern is consistent with diffusion-limited vector transport in densely packed 3D systems, where extracellular matrix composition and cell density constrain penetration depth [34, 35]. However, because GFP fluorescence represents the downstream consequence of vector delivery and transgene expression rather than vector localization itself, however, the observed radial pattern should not be interpreted as a direct measurement of physical AAV penetration. The peripheral distribution may reflect differences in vector exposure, cellular composition, or downstream transgene expression within the spheroids. In contrast, PDTCs demonstrated a more homogeneous distribution of GFP fluorescence across the investigated tissue regions, consistent with differences in tissue organization and vector exposure between the two model systems. Notably, this effect was not associated with preferential vascular localization, indicating that improved penetration is likely driven by preserved tissue microarchitecture rather than vascular entry routes [34, 36].

Although overall GFP-based readouts were comparable between PDNS and PDTC, PDTC demonstrated substantially greater interpatient variability. Several patient-derived samples showed little or no detectable GFP expression despite identical experimental conditions. This observation highlights the profound biological heterogeneity of GBM and aligns with previous reports demonstrating that tumor subtype, receptor expression, and microenvironmental composition influence susceptibility to experimental therapies, including viral vectors [9, 37–39]. The different degree of interpatient variability observed between PDNS and PDTCs further illustrates the trade-off between experimental control and preservation of patient-specific biology. The comparatively lower variability of PDNS may facilitate controlled comparisons of vector characteristics and increase statistical sensitivity during initial screening. However, this apparent reproducibility may partly result from selection and adaptation during sphere establishment and expansion, potentially reducing the diversity of cellular states represented in the original tumors. Conversely, the greater variability observed in PDTCs complicates statistical comparisons but may retain biologically relevant interpatient heterogeneity in cellular composition, tissue architecture, and microenvironmental context. Accordingly, variability in PDTCs should not automatically be regarded as experimental noise but may itself represent an important parameter when evaluating the robustness of candidate vectors across heterogeneous GBM tissues. This reinforces the necessity of multi-patient validation frameworks when evaluating gene therapy platforms for GBM.

Occasional GFP-positive cells were also observed in adjacent non-neoplastic brain tissue, indicating GFP expression following AAV exposure can occur in cells within tumor-adjacent tissue under the experimental conditions used. This observation is consistent with the broad CNS tropism reported for naturally occurring AAV serotypes [40, 41] and underscores the importance of considering off-target transduction during the development of GBM-directed gene therapies. Because the cellular identity of this preliminary observation of GFP-positive cells was not determined in the present study, no conclusions can be drawn regarding the preferential transduction of neuronal, glial, vascular, or immune cell populations. Characterization of transduced cells in tumor-adjacent brain tissue will therefore be important for assessing cellular specificity and potential off-target effects of candidate vectors.

SOX2 expression also varied with serotype and EGF conditions in PDNS, with a significant increase observed following AAV6 treatment in the absence of EGF. However, these findings do not establish that AAV exposure directly alters tumor-cell state. The observed differences may reflect selective transduction or survival of pre-existing populations, changes in cellular composition, or culture-dependent adaptations [29, 42–44]. Accordingly, the present data support an association between AAV treatment, culture conditions, and SOX2 expression but do not establish a causal biological effect of AAV exposure. Longitudinal or single-cell analyses would be required to resolve these possibilities.

Several limitations should be acknowledged. First, vector dose escalation could not be performed in PDTC because of limited tissue availability, preventing direct dose-matched comparisons between the two model systems. Second, the marked interpatient heterogeneity characteristic of GBM reduced statistical power and limited generalization of individual observations, although this variability simultaneously reflects a biologically relevant patient-specific difference of the PDTC model. Third, the additional molecular and cellular analyses were performed in relatively small subsets of PDNS. Vector genome quantification supported the serotype-dependent differences observed by fluorescence analysis but showed considerable inter-sample variability, while the flow-cytometric analysis was limited to two independent experiments and therefore remains exploratory. Neither analysis resolves the complete sequence of events underlying productive AAV-mediated gene expression. Vector genome measurements do not distinguish cellular entry, intracellular trafficking, genome persistence, or genome processing, whereas GFP fluorescence additionally depends on transcription and translation. Finally, although PDTC preserve important features of the tumor microenvironment, they remain ex vivo models that cannot fully reproduce systemic immune responses, vascular dynamics, or long-term tumor evolution.

In conclusion, our findings demonstrate that the experimental model is a critical determinant of AAV-mediated gene delivery in GBM. PDNS provide a comparatively controlled patient-derived system for vector screening and investigation of tumor-cell-state-dependent effects, whereas PDTCs preserve patient-specific tissue architecture and microenvironmental features that influence vector distribution and cellular responses. Together, these findings support a question-driven use of complementary patient-derived models for preclinical evaluation of AAV-based gene delivery in GBM.

## Electronic Supplementary Material

Below is the link to the electronic supplementary material.

Supplementary Material 1

## Data Availability

The datasets generated and analyzed during the current study are available from the corresponding author on reasonable request. Access to de-identified data may be subject to institutional ethics approval and applicable data protection regulations due to the use of human patient-derived material.
