# Supplementary material for: Patient-derived tissue cultures complement neurospheres for preclinical evaluation of AAV-mediated gene delivery in glioblastoma
Source: J Neurooncol. 2026 Sep 22;179(3):93. doi: 10.1007/s11060-026-05807-w (PMC13597561; doi:10.1007/s11060-026-05807-w)
Supplement: Supplementary file 1 [file 11060_2026_5807_MOESM1_ESM.docx]

**Supplement**

**Supplementary Figure 1. Effect of culture media on tumor cell proliferation and apoptosis in patient-derived GBM specimens.** Four independent patient-derived tumor specimens were cultured under three different media conditions to assess tissue viability after 4 days in vitro. Cellular proliferation was evaluated by Ki67 immunostaining, and apoptosis was assessed by cleaved PARP (cPARP) expression. For one specimen, only three culture conditions were available; therefore, a day 0 baseline control was not included for this sample. Quantitative analysis demonstrated an overall reduction in proliferative activity across all culture conditions compared with baseline. In parallel, an increase in apoptotic activity was observed; however, the magnitude of effects on proliferation and apoptosis varied between individual patient specimens, indicating inter-sample heterogeneity in culture response. Statistical significance was assessed using the Wilcoxon signed-rank test (p < 0.05).

**Supplementary Figure 2. Semi-quantitative assessment of CD44 expression in patient-derived GBM cultures.** CD44 expression in tissue cultures was evaluated using a semi-quantitative pathological scoring system commonly applied in histopathological analyses. Representative microscopic fields were categorized into four groups: negative (-), low (+), moderate (++), and high (+++) CD44 expression based on staining intensity and distribution of nativ (61 pictures), PDTC (288 pictures) and PDNS (187 pictures). Overall analysis indicated an increased frequency of CD44-positive fields across both culture conditions compared with baseline classification. However, individual patient-derived samples (#8 and #15) demonstrated marked variability in CD44 expression patterns, highlighting culture-dependent heterogeneity and inter-sample differences in marker distribution. These observations underscore dynamic cellular adaptation under ex vivo culture conditions.

**Supplementary Figure 3. Longitudinal analysis of AAV transduction and methodological controls in PDNS.** (A) GFP expression was monitored by live confocal microscopy for up to 14 days following AAV transduction. AAV6-mediated transduction resulted in higher GFP expression at early time points compared with AAV2. In EGF-supplemented cultures, GFP intensity relative to spheroid size decreased at later time points (day 12–14), whereas cultures maintained without EGF exhibited lower initial transduction but more stable GFP expression throughout the observation period. As EGF supplementation substantially affected spheroid growth kinetics, differences in spheroid diameter between culture conditions were monitored and considered during data interpretation. (B) Evaluation of transport medium composition on spheroid viability and transduction efficiency. Serum supplementation during tissue transport was assessed as a potential methodological confounder. Propidium iodide (PI) staining and GFP expression demonstrated no apparent differences in spheroid viability or AAV-mediated transduction between serum-containing and serum-free transport conditions. Representative data from all biological replicates are shown (n=2–3 independent specimens). (C) Representative flow-cytometric analysis of a patient-derived specimen for which sufficient PDNS were available to assess all experimental conditions. Following gating for singlets and viable events, GFP expression was assessed in populations defined by the stemness-associated markers CD133, Nestin, and SOX2. PDNS treated with AAV6 in the presence or absence of EGF are shown in the lower panels. Purple events indicate GFP-positive events, whereas red and blue events distinguish marker-defined cellular populations. The same gating strategy and color coding were applied across conditions. Flow-cytometric analysis was performed as an exploratory assessment complementing the immunohistological characterization of marker-defined populations. Three independent experiments yielded sufficient PDNS for flow-cytometric evaluation; one additional experiment was excluded because of insufficient cellular viability. (D) Cell type-specific transduction analysis of stem-like GBM populations. Co-immunofluorescence staining identified GFP-positive cells expressing Survivin (upper panel) and Olig2 (lower panel). Under EGF-supplemented conditions, GFP-positive Survivin- and Olig2-expressing cells were rarely detected, whereas limited transduction of these populations was observed in cultures maintained without EGF. Quantification of marker-defined (CD44, GFAP, Nestin, SOX2) cellular populations revealed condition-dependent trends following AAV exposure; however, none of these differences reached statistical significance.

**B**

**A**

**Supplementary Figure 4. CD44 expression and representative immunofluorescence images following AAV transduction in PDTC and PDNS.** (A) CD44 expression was assessed following AAV2 supplementation and AAV6 transduction under EGF-supplemented and EGF-deprived conditions in PDTCs and PDNS. CD44 expression remained overall stable across culture systems and treatment conditions. A non-significant reduction in CD44-positive cell populations was observed in PDNS following AAV6 transduction, independent of EGF supplementation, whereas no changes were detected in PDTCs. (B) Representative immunofluorescence images showing co-localization of GFP and stem cell–associated markers in PDTCs (upper row) and PDNS (lower row) following AAV transduction. Images illustrate culture-dependent differences in marker distribution and transgene expression.
